# Congenital anomalies in Santa Catarina: case distribution and trends in 2010–2018

**DOI:** 10.1590/1984-0462/2022/40/2020331

**Published:** 2021-10-04

**Authors:** Bruna Muraro Vanassi, Gabriel Cremona Parma, Vivyane Santiago Magalhaes, Augusto César Cardoso dos Santos, Betine Pinto Moehlecke Iser

**Affiliations:** a Universidade do Sul de Santa Catarina, Tubarão, SC, Brazil.; b Secretaria de Vigilância em Saúde, Ministério da Saúde, Brasília, DF, Brazil.

**Keywords:** Congenital abnormalities, Genetic diseases, inborn, Health information systems, Vital statistics, Epidemiologic studies, Anormalidades congênitas, Doenças genéticas inatas, Sistemas de informação em saúde, Estatísticas vitais, Estudos epidemiológicos

## Abstract

**Objective:**

To evaluate the distribution of cases of congenital anomalies in the state of Santa Catarina by health macro-region, to determine the frequency according to maternal and neonatal variables, to estimate the related mortality, and the trends in the period 2010–2018.

**Methods:**

An ecological time-series study with secondary data on congenital anomalies and the sociodemographic and health variables of mothers and newborns living in Santa Catarina, from 2010 to 2018. For temporal trend analysis, generalized linear regression was performed using the Prais-Winsten method with robust variance.

**Results:**

The average prevalence of congenital anomalies in the period was 8.9 cases per 1,000 live births, being 9.4 cases by 1,000 live births in 2010 and, in 2018, 8.2/1,000. The trend remained stable in the analyzed period. The major malformations were musculoskeletal, hip, and foot malformations, with a proportion ≥30%. There was a higher prevalence of congenital anomalies in low birthweight, preterm, male livebirths with Apgar≤7, born by cesarean section, mothers of older age (≥40 years), and less educated (less than eight years of study). Infant mortality due to congenital malformations was 2.6 deaths/1,000 live births, representing about 25.8% of the total infant deaths in the period.

**Conclusions:**

The frequency of congenital anomalies and the mortality with anomalies was stable in the studied period in Santa Catarina. The presence of anomalies was associated with low birth weight, prematurity, and low Apgar score. The highest proportion of congenital anomalies was in the musculoskeletal system.

## Resumo

**Objetivo:**

Avaliar a distribuição dos casos de anomalias congênitas no estado de Santa Catarina por macrorregião de saúde, determinar a frequência segundo fatores da mãe e do produto da gestação e estimar a mortalidade relacionada e a tendência no período de 2010–2018.

**Métodos:**

Estudo ecológico de série temporal, com dados secundários sobre anomalias congênitas e variáveis sociodemográficas e de saúde de mães e recém-nascidos residentes em Santa Catarina, no período de 2010 a 2018. Para análise de tendência temporal, foi utilizada a regressão linear generalizada pelo método de Prais-Winsten com variância robusta.

**Resultados:**

A prevalência média de anomalias congênitas no período foi de 8,9 casos a cada 1.000 nascidos vivos, sendo 9,4 casos a cada 1.000 em 2010 e 8,2 a cada 1.000 em 2018. A tendência manteve-se estável no período analisado. As principais malformações foram as osteomusculares, de quadril e de pés, com proporção ≥30%. Verificou-se prevalência maior de anomalias congênitas nas crianças de baixo peso, prematuras, de sexo masculino e Apgar≤7 e nos nascidos de cesariana, de mães de maior idade (≥40 anos) e menor escolaridade (menos de oito anos de estudo). A mortalidade infantil por malformações congênitas foi de 2,6 óbitos/1.000 nascidos vivos, representando 25,8% do total de óbitos infantis no período.

**Conclusões:**

Houve estabilidade na frequência e na mortalidade associada às anomalias congênitas em Santa Catarina de 2010 a 2018. A ocorrência dessas condições foi associada aos nascidos de baixo peso, pré-termo e baixo escore de Apgar. A maior proporção de anomalias congênitas foi do sistema osteomuscular.

## INTRODUCTION

The term congenital anomalies (CA) refers to a set of functional and/or morphological changes, including malformations, deformations, and disruptions, which occur in intrauterine life.^1^ These conditions can be detected during or after pregnancy and have a genetic relationship, environmental or unknown.^1^ For approximately half of the cases, it is not possible to establish a single etiological factor, and there may be multiple associated causes.^2^

The studies by Martin et al.^3^ and Kliegman et al.^4^ cite the worldwide incidence of congenital malformations (CM) between 2 and 2.5%. These conditions can contribute to long-term disability, which can significantly impact individuals, families, health systems, and society.^1^ It is estimated that 303,000 newborns (NB) die within four weeks of birth each year in the world, due to CA.^2^

In Brazil, CA represent the second cause of death in children under 1 year of age, in all regions of the country, corresponding to 22% of infant deaths, second only to prematurity.^5^ Many CA are preventable at different levels, such as vaccination, adequate intake of folic acid or iodine, and prenatal.^2^ Alerting professionals who monitor maternal and child health is essential for reducing child mortality in the country, with possible implication in the development of healthy and productive individuals for the society.^6^

Santa Catarina belongs to the South Region of Brazil, a federative unit (FU) that has the lowest absolute population (total number of inhabitants) in the region, compared to the adjacent states — Paraná and Rio Grande do Sul. According to the population estimate of the Brazilian Institute of Geography and Statistics (*Instituto Brasileiro de Geografia e Estatística* – IBGE), in 2019 the population of Santa Catarina was 7,164,788 inhabitants.^7^ It is considered the group by FU with the longest life expectancy in Brazil and has an average human development index of 0.774, standing in the range of high human development, between 0.700 and 0.799.^7^ The territorial division made available by the Live Birth Information System (*Sistema de Informações sobre Nascidos Vivos* – SINASC) includes seven health macro-regions: South, North and Northeast plateau, Midwest and Santa Catarina mountain range, great West, Greater Florianópolis, Foz do Rio Itajaí, and Alto Vale do Itajaí.

In view of the complexity of the theme and the little local knowledge about the occurrence of the problem and related factors, the study sought to evaluate the distribution of CA cases in the state of Santa Catarina by health macro-region and to determine the frequency of cases according to factors of the mother and the product of pregnancy, in addition to estimating related mortality and its trend in the 2010–2018 period.

## METHOD

An ecological study of temporal trend was carried out, considering all cases of CA registered in Santa Catarina in the years 2010–2018. The investigation focused on the occurrence of CA, a measure of general frequency, by categories and constant diagnosis of the International Statistical Classification of Diseases and Related Health Problems (ICD-10), codified in chapter XVII, “Congenital malformations, deformities and anomalies chromosomal (Q00 – Q99)”. The data necessary for the research were accessed through the TABNET virtual platform, from the Informatics Department of the Unified Health System. The data on the frequency of anomalies came from the Declaration of Live Birth, in SINASC, and, for infant deaths related to anomalies, derived from the Mortality Information System (*Sistema de Informações sobre Mortalidade* – SIM), considering the “congenital anomaly”, ICD-10 Q00–Q99, as the underlying cause.

The microdata required for the study were collected in an aggregated form and tabulated with the aid of Microsoft Excel. As this is a research with secondary and aggregated data from information systems, without personal identification, open to public consultation, this study did not need to be evaluated by the Research Ethics Committee.

The occurrence of CA was described according to the mother's characteristics, gestational and NB data, and considering the health macro-regions of Santa Catarina according to the mother's home address. Prevalence and mortality were calculated for every 1,000 live births (LB) in the years 2010 to 2018.

The variables presence of CA (yes or no) and type of anomaly were evaluated, according to the ICD-10 coding:

- CM of the nervous system (Q00–Q07);
- CM of the circulatory system (Q20–Q28);
- cleft lip and cleft palate (Q35–Q37);
- absence, atresia, and stenosis of the small intestine (Q38–Q41.8) and CM of the digestive tract (Q45–Q48);
- malformations of the genitourinary system (Q60–Q64) and undescended testicle (Q50–Q53);
- malformations and congenital deformations of the musculoskeletal system, including congenital deformities of the hip and feet (Q65–Q79);
- chromosomal abnormalities — not elsewhere classified (Q90–Q99);
- other CM, along with hemangioma (D18.0) and lymphangioma (D18.1).

As for the pregnancy data, its duration was categorized as preterm (<37 weeks), term (37 to 41 weeks), and post-term (≥42 weeks). The type of delivery was classified as vaginal or cesarean; the gender of the newborn in male or female; birth weight in grams, categorized as ≤ 2,499, from 2,500 to 3,999 or ≥4,000; and 5-minute Apgar score, considered low when from 0 to 7 and appropriate when from 8 to 10. For the mother's data, age was considered, in years (from 10 to 19 years, from 20 to 29, from 30 to 39, and ≥40 years); education, in the categories none, from 1 to 7, from 8 to 11, and ≥12 years of study; and race/color (white, black, yellow, and indigenous). Ignored data were excluded from the specific tabs.

Prevalence rates were calculated, according to the study variables, through the number of anomaly cases in each year and the total number of LB in the same categories × 1,000. The proportions of each type of CA were also considered, according to the system or organ affected, in relation to the total number of registered cases.

Proportional mortality was also calculated, considering the number of infant deaths (up to 1 year of life) that had diseases of Chapter XVII of ICD-10 as a basic cause to registered infant deaths × 100, and the infant mortality rate, when in the denominator, the total number of LB for the same place and period was pointed out.

For temporal trend analysis, generalized linear regression was used by the Prais-Winsten method, with robust variance and adjustment for autocorrelation proposed by Durbin and Watson, with values close to 2 being expected as indicative of the absence of serial autocorrelation. The positive/negative slope value (β) represents the mean annual increase/decrease in rates for each year analyzed. Based on it, the trend was also assessed, whether it was stationary (p> 0.05), declining (p <0.05 and negative regression coefficient) or ascending (p <0.05 and positive regression coefficient), in each category of variables studied. The level of statistical significance was 5%.

The mean of the anomaly rates in the study period was estimated, as well as their respective 95% confidence intervals (95% CI), allowing the comparison of rates according to the characteristics of interest.

To map the cases, the QGIS software was used, linking the prevalence data with the health macro-regions of the state of Santa Catarina. The cartographic data used were the shape maps of the official Brazilian cartographic system, of the municipalities and the state of Santa Catarina, made available by IBGE, in addition to the regional health regions defined by Santa Catarina.

## RESULTS

**Figure 1:**
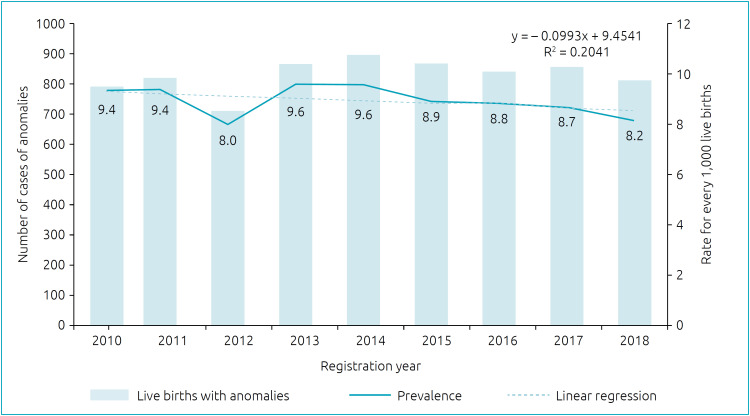
Time trend of the occurrence of congenital anomaly in Santa Catarina from 2010 to 2018.

**Figure 2:**
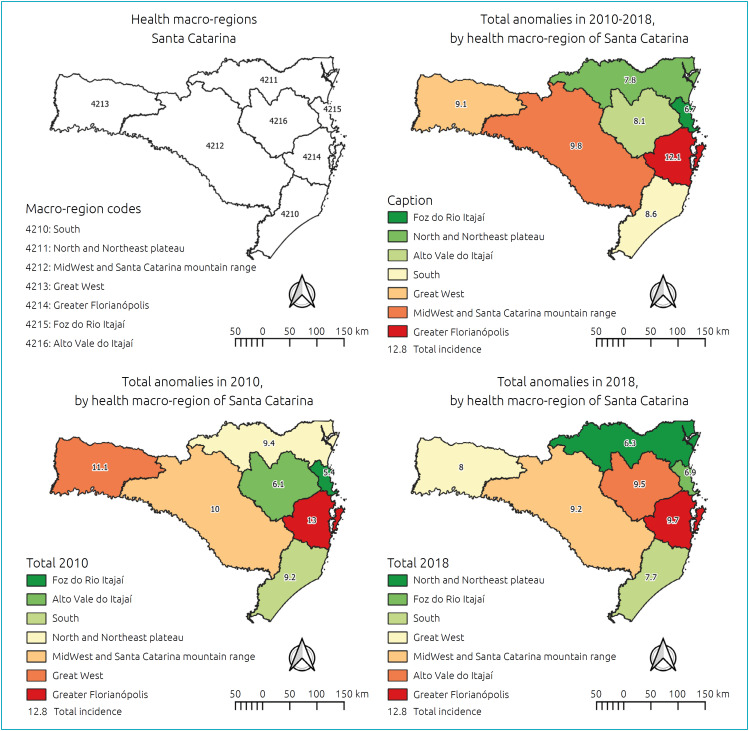
Geographic distribution of the occurrence of congenital anomalies according to the health macro-regions of Santa Catarina, in the years 2010 and 2018 and in the accumulated period of 2010–2018.

Among 834,451 LB in Santa Catarina, from 2010 to 2018, 7,464 cases of CA were recorded — mean prevalence of 8.9 cases per 1,000 LB in the period, regardless of the type registered. In 2010, 9.4 cases were identified for every 1,000 LB, and in 2018, 8.2 for every 1,000 (Figure 1). This apparent reduction was not uniform over the years or among the macro-regions, showing a tendency for stability in the state (Table 1). Among the macroregions, the southern, northern, and northeastern plateau regions showed a significant downward trend in the period. Greater Florianópolis was noteworthy (Figure 2), with a higher prevalence than the other regions in all years, except in 2012, a year in which there was a frequency of 7.4 cases/1,000 LB and was surpassed by the Midwest and Santa Catarina mountain range regions (10 cases/1,000 LB).

**Table 1:** Prevalence* of congenital anomalies in Santa Catarina according to the year of analysis and characteristics of the mother in the 2010–2018 period.

Categories/year		Annual rate*									Total 2010–2018		Trend	p-value
		2010	2011	2012	2013	2014	2015	2016	2017	2018	Mean	95%CI	Coefficient β#	
Santa Catarina		9.4	9.4	8.0	9.6	9.6	8.9	8.8	8.7	8.2	9.0	8.5–9.4	−0.10	0.116
Macro-region														
	South	9.2	8.3	8.7	9.9	9.0	7.8	8.4	8.3	7.7	8.6	8.0–9.1	−0.15	0.035
	North/Northeast plateau	9.4	9.2	7.0	7.8	8.2	7.9	7.9	6.9	6.3	7.8	7.1–8.6	−0.31	0.006
	Midwest and mountain range	10.0	11.1	10.0	9.9	8.7	9.7	9.4	9.9	9.2	9.8	9.3–10.3	−0.14	0.050
	Great West	11.1	9.1	9.5	8.5	7.9	9.1	10.5	8.7	8.0	9.1	8.3–9.9	−0.25	0.178
	Great Florianópolis	13.0	13.8	7.4	13.1	16.4	12.1	12.0	11.3	9.7	12.1	10.1–14.0	−0.22	0.382
	Foz do Rio Itajaí	5.4	4.2	5.8	8.7	7.9	8.9	5.4	6.6	6.9	6.7	5.4–7.9	0.20	0.300
	Alto Vale do Itajaí	6.1	8.0	7.8	9.4	8.0	6.9	7.6	9.0	9.5	8.1	7.2–8.9	0.31	0.043
Age of the mother (years)														
	10 to 19	9.8	9.5	7.6	9.8	10.2	8.8	8.6	8.2	6.3	8.8	7.8–9.7	−0.32	0.044
	20 to 29	8.5	8.0	7.5	9.3	8.7	7.7	7.8	7.9	7.4	8.1	7.6–8.6	−0.10	0.083
	30 to 39	9.9	10.9	8.4	9.2	10.1	10.4	9.9	9.4	8.7	9.6	9.0–10.3	−0.09	0.396
	≥40	17.7	18.1	16.6	19.9	17.9	13.4	15.5	15.7	18.9	17.1	15.6–18.6	−0.12	0.670
Mother's level of education (years of study)														
	None	8.4	15.6	11.7	34.5	22.6	14.3	0.0	17.2	18.2	15.8	8.5–23.2	0.24	0.833
	1 to 7	10.3	10.7	8.1	10.2	9.6	9.9	10.3	10.9	8.9	9.9	9.2–10.6	0.00	0.987
	8 to 11	8.8	8.5	7.8	9.3	9.7	8.4	7.9	7.9	7.4	8.4	7.8–8.9	−0.16	0.026
	≥12	9.3	9.7	8.2	9.7	9.4	9.6	10.1	9.3	9.5	9.4	9.0–9.8	0.06	0.279
Mother's color/race														
	White	9.2	9.2	8.1	9.5	9.6	8.8	8.9	8.8	8.1	8.9	8.5–9.3	−0.08	0.175
	Black (black+brown)	12.1	10.1	7.8	10.8	9.7	10.3	8.6	9.7	9.5	9.8	8.9–10.8	−0.17	0.290
	Yellow	0.0	27.4	0.0	0.0	18.2	0.0	0.0	0.0	13.4	6.6	0–14.6	−0.83	0.517
	Indigenous	23.4	26.7	11.3	21.3	11.4	9.8	3.3	6.3	0.0	12.6	5.5–19.7	−3.08	<0.001

* Number of births with anomalies/total number of live births × 1,000; 95%CI: 95% confidence interval

# beta regression coefficient.

Regarding the mother's characteristics, there was a higher prevalence of anomalies for NB of mothers aged 40 years old or older, in all years, and lower education (less than eight years of study). There was a tendency to reduce the occurrence of CA in newborns in the period for adolescent mothers (up to 19 years of age) and for those with schooling from eight to 11 years of study. According to race/color, indigenous women had the highest mean frequency of CA, and yellow women the lowest, but with great variation between years. The trend was decreasing only for the indigenous race (Table 1).

Regarding birth data (Table 2), there was a higher occurrence of CA in low birth weight (29.6 per 1,000 LB, 95%CI 28.3–30.9) and in preterm infants (21.6 for every 1,000 LB; 95%CI 19.4–24.5). With regard to the type of delivery, there was a higher prevalence in those born by cesarean section (10.1 per 1,000 LB) than by vaginal births (6.9 per 1,000 LB), with higher rates of CA in Greater Florianópolis. The evaluation by gender shows a prevalence of 10.5 cases for every 1,000 LB boys and 7.5 for every 1,000 LB girls, with a decreasing trend for males. In relation to the 5-minute Apgar, the prevalence was eight times higher for children with Apgar≤7 (64/1,000; 95%CI 57.8–70.3) compared to those with Apgar from 8 to 10 (7.7/1,000; 95%CI 7.3–8.2), although with a downward trend in the period.

**Table 2:** Prevalence* of congenital anomalies in Santa Catarina according to the year of analysis and the characteristics of the pregnancy and the newborn during the period from 2010 to 2018.

Categories/year		Annual rate*									Total 2010–2018		Trend	p-value
		2010	2011	2012	2013	2014	2015	2016	2017	2018	Mean	95%CI	Coefficient β#	
Birth weight (g)														
	≤2,499	31.8	30.7	26.4	32.1	29.5	29.7	29.4	28.4	28.5	29.6	28.3–30.9	−0.24	0.156
	2,500 to 3,999	7.4	7.5	6.4	7.7	7.9	7.1	7.4	7.1	6.6	7.2	6.9–7.6	−0.05	0.383
	≥4,000	8.2	8.0	6.8	7.8	9.1	7.9	10.9	5.6	4.6	7.6	6.2–9.1	−0.27	0.380
Type of delivery														
	Vaginal	6.2	6.5	5.9	7.4	7.9	6.9	5.8	6.0	5.6	6.5	5.9–7.1	−0.08	0.479
	Cesarean	8.9	9.1	9.3	11.0	10.8	10.4	11.1	10.6	10.0	10.1	9.5–10.8	0.17	0.171
Gestation length														
	Pre-term^a^	29.1	23.1	16.6	23.2	22.4	21.2	20.1	20.1	22.1	21.6	19.4–24.5	−0.63	0.313
	Term^b^	7.8	8.1	7.0	8.0	8.2	7.4	7.5	7.4	6.5	7.5	7.1–7.9	−0.11	0.086
	Post-term^c^	2.2	5.6	5.6	7.7	6.7	6.5	5.4	4.4	7.5	5.7	4.4–7.0	0.50	0.180
Gender of the newborn														
	Male	10.6	11.1	9.3	11.2	11.6	10.2	9.5	9.3	8.7	10.2	9.4–10.9	−0.23	0.124
	Female	7.8	7.4	6.5	7.8	7.4	7.5	7.8	7.8	7.3	7.5	7.2–7.8	0.03	0.620
5-minute Apgar														
	0 to 7	63.2	71.5	61.1	82.8	60.9	57.6	61.7	58.4	59.1	64.0	57.8–70.3	−1.47	0.024
	8 to 10	8.2	8.1	6.8	8.1	8.5	7.8	7.7	7.6	7.0	7.7	7.3–8.2	−0.09	0.149

* Number of births with anomalies/total number of live births × 1,000; 95%CI: 95% confidence interval

# beta regression coefficient

a <37 weeks of gestation

b 37–41 weeks

c ≥42 weeks.

As for the type of anomaly, the musculoskeletal movements of the hip and feet predominated in the period, representing 34% of the total and prevalence of 3.1 cases per 1,000 LB, followed by malformations of the genitourinary system and undescended testicle (13.4% and prevalence of 1.2/1,000) and other CM, including hemangioma and lymphangioma (12.1% and 1.1 cases/1,000). Table 3 shows the mean occurrence of CA according to type in the analyzed period. There were no differences over time regarding this criterion (trend data not shown).

**Table 3:** Proportion and rates of anomalies recorded in Santa Catarina in total, during the 2010–2018 period, according to the type, group or system involved.

Type of anomaly	Births with anomalies	%	Mean prevalence*	95%CI
Musculoskeletal system	2,537	34.0	3.1	2.82–3.30
Genitourinary∞	998	13.4	1.2	1.05–1.36
Others¤	902	12.1	1.1	1.02–1.15
Circulatory system	806	10.8	1.0	0.86–1.08
Nervous system	779	10.4	0.9	0.87–1.00
Cleft lip and palate	602	8.1	0.7	0.68–0.77
Chromosomes	494	6.6	0.6	0.53–0.67
Digestive system #	352	4.7	0.4	0.39–0.45
Total	6,022	100	8.9	8.49–9.42

* Number of births with anomalies/total number of live births × 1,000; 95%CI: 95% confidence interval

# in malformations of the digestive system, including absence, atresia, and stenosis of other parts of the small intestine

∞ malformations of the genitourinary system, including undescended testicle

¤ in the category others, comprising minor congenital malformations, including hemangioma.

Among the types highlighted according to the state's macro-regions, anomalies of the nervous system and spina bifida gained prominence in the north and northeast plateau (prevalence of 1.5/1,000), those of the circulatory system in Greater Florianópolis (2.5/1,000), cleft lip and palate in the Alto Vale do Itajaí and the north and northeast plateau (with a prevalence of 1/1,000), and the others (digestive, genitourinary, musculoskeletal, chromosomal, and others) in Greater Florianópolis, with higher frequencies considering the state as a whole (Table 4).

**Table 4:** Distribution of types of anomaly according to the macro-regions of the state of Santa Catarina, during the 2010–2018 period.

Prevalence*	Nervous system	Circulatory system	Cleft lip and palate	Digestive#	Geniturinary∞	Musculoskeletal	Chromosomes	Others¤
South	0.14	0.14	0.11	0.06	0.15	0.37	0.10	0.10
North/Northeast plateau	1.48	1.41	1.03	0.60	1.13	4.08	0.62	1.54
Midwest and mountain range	0.67	0.60	0.54	0.31	0.80	2.46	0.36	0.76
Great West	0.77	0.41	0.67	0.37	1.03	2.88	0.66	0.86
Great Florianópolis	1.36	2.51	1.00	0.73	3.31	4.91	0.91	2.36
Foz do Rio Itajaí	0.49	0.41	0.32	0.17	0.56	1.39	0.38	0.56
Alto Vale do Itajaí	1.16	1.02	1.03	0.55	1.17	4.22	0.78	1.32
Mean of the years	0.87	0.93	0.67	0.40	1.16	2.90	0.54	1.07

* Number of births with anomalies/total number of live births × 1,000

# in malformations of the digestive system, including absence, atresia, and stenosis of other parts of the small intestine

∞ malformations of the genitourinary system, including undescended testicle

¤ in the category others, comprising minor congenital malformations, including hemangioma.

Infant mortality due to CA, represented by chapter XVII of ICD-10, was 2.6 deaths due to anomalies per 1,000 LB, being slightly higher in the macro regions of Foz do Rio Itajaí and Alto Vale do Itajaí, without significant differences. Mortality ranged from 2.3/1,000 LB in 2015 and 2016 to 3.1/1,000 LB in 2011, with a stable trend in the period. Infant mortality due to CA corresponded to 25.8% of the total infant deaths in the period, varying from 23.5% in 2015 to 27.9% in 2013 and 2017.

## DISCUSSION

This study aimed to investigate data on the prevalence and distribution of CA in the state of Santa Catarina from 2010 to 2018. The average rate for the period was 8.9 cases per 1,000 LB. In an analysis of the historical series from 2006 to 2017, the national prevalence ranged from 6.19 cases/1,000 LB in 2006 to 8.62 cases/1,000 LB in 2017.^8^ In the period from 2005 to 2014, there were 1,386,803 births from mothers living in Rio Grande do Sul, 12,818 (0.92%) with CA, which corresponds to the general mean rate of 9.2 per 1,000 cases (95%CI 8.4–10.3).^1^ Thus, there is similarity between the prevalence of Santa Catarina to the national data and also to those of Rio Grande do Sul, the adjacent state.

Data released in the Epidemiological Bulletin of Brazil, in March 2020, showed an overall increase of 25% in the record of CA in SINASC in the period from 2010 to 2018 of the FU analyzed, with the concentration of the highest prevalences in the states of the Southeast and South regions,^8^ which may be associated to greater detection of cases in these regions due to a more active and structured surveillance. Among the macroregions of the state of Santa Catarina, Greater Florianópolis had the highest rates for most CA, which may be linked to a possible concentration of notifications in large urban centers and places of reference for high-risk childbirth.^9^ ^,^ ^10^ Pregnancies of older women have a higher incidence of diabetes, high blood pressure and, consequently, a higher probability of perinatal complications, such as abortion, CA, pre-eclampsia, eclampsia, premature births, among others.^11^ For better monitoring of these pregnancies, considered of high risk, there is a need for specialized health services, with the performance of prenatal exams for morphological evaluation and genetic studies — if necessary —, in addition to monitoring these at-risk pregnant women.^12^ ^-^ ^15^

The most prevalent CA in Brazil were musculoskeletal defects (1.31 cases per 1,000 LB), followed by congenital heart defects (0.81/1,000), oral clefts (0.61/1,000), and neural tube defects (0.45/1,000).^8^ The predominance of musculoskeletal malformations is also visible in the state of Santa Catarina and may be related to the ease of diagnosis, as these are macrosomal malformations, visible and detectable on physical examination, diagnosed early in the immediate postnatal period.^6^ ^,^ ^9^ In other studies, the prevalence of involvement of the musculoskeletal system was also observed in the cities of Fortaleza (Ceará) (30%),^16^ São Paulo (São Paulo) (29.9%)^6^, São Luís (Maranhão) (48 %)^17^, and in first world countries, such as the United States and some countries in Europe.^2^ ^,^ ^18^

Still, the higher prevalence of anomalies in low birth weight, preterm and low Apgar births, in addition to agreeing with other studies already published, such as in Rio Grande do Sul^1^ and São Paulo,^6^ suggests a relationship between the presence of CA with a risk gradient and critical state of the newborn. The relationship verified with cesarean delivery may also indicate that there was a previous diagnosis, during prenatal care, of a malformation that led to the choice for a surgical delivery, indicated in some high-risk situations.^19^

The higher mean rate of anomalies in mothers with low education may reflect greater difficulties that women with little training have to perform prenatal care and timely diagnosis, in addition to possible nutritional deficiencies, greater use of alcohol and other drugs, or even lack of information and search for adequate care.^12^ The higher frequency of CA in children of mothers aged 40 years old or older corroborates the study carried out in Rio Grande do Sul,^1^ which also showed higher rates of CA among mothers aged older than or equal to 40 years. Advanced age stands out for being an important risk factor recognized in the context of CA, particularly because of Down Syndrome.^11^ ^,^ ^20^

In the regions of Foz and Vale do Itajaí, due to the occurrence of cleft lip and palate and the higher mortality rate due to CA, it was found that the productive system of the place is based on the cultures of onion, tobacco, and rice, in addition to pigs and poultry.^21^ It is worth investigating the role of these crops, the method of planting and the pesticides used as possible causes of malformations with significant occurrence in the region. Studies show that pesticides can affect the male reproductive system of animals and also embryo-fetal development after intrauterine exposure, including CM.^22^ ^,^ ^23^

Some studies show a higher occurrence of CM due to the proximity of the residences to the cultivation areas.^24^ ^-^ ^26^ The greatest association found in the state of Paraná, FU adjacent to Santa Catarina, was related to the malformation classified as undescended testicle.^27^ As many pesticides are endocrine disruptors, it is suspected that they influence the sexual differentiation of the fetus and other gender hormone-dependent outcomes.^27^ There is evidence about the association between cryptorchidism,^28^ hypospadias,^29^ and exposure to pesticides, pointing out that such problems are related to the fluctuation of female and male hormones in the gestational period, which are directly influenced by environmental conditions.^27^

Specific mortality due to CA in Santa Catarina showed stability in the period evaluated, in contrast to the drop in global infant mortality observed in Brazil and in the world, a fact that can be further explored in future studies. In the period from 1990 to 2016, the infant mortality rate fell from 93 to 41 deaths per 1,000 LB, representing a 56% decline.^30^ In Brazil, although CA represent the second cause of mortality in children under 5 years of age,^31^ between 1990 and 2014 infant mortality decreased by 70%, from 47.1 to 14.1 deaths per 1,000 LB.^5^ The data from Santa Catarina investigated in this study, however, indicate stability of mortality related to CA, a point that can be deepened in future works.

A limitation is the collection of secondary data, depending on the quality of the data in the information systems, with some information ignored. There was also no linkage of data from SINASC and SIM, which may underestimate the frequency of CA, especially for those not detectable at birth.^9^ As a strong point, we did not find recently published studies addressing the frequency of CA in the state of Santa Catarina, which allows us to infer that this work should contribute to a better understanding of this phenomenon in the region, and can subsidize specific diagnostic actions, in addition to strengthening prenatal care as a whole.

The prevalence found was similar to data from Brazilian and regional literature. There was stability in the frequency and mortality of CA in Santa Catarina from 2010 to 2018. The occurrence of anomalies was associated with those born with low weight, preterm, and low Apgar. The highest proportion of CA was from the musculoskeletal system.

It is considered important to improve the system for capturing and registering CA visible at birth in the state of Santa Catarina, through the structuring of an active surveillance system that includes constant monitoring of the disease and the training of health care teams, with a view to guiding health policies and interventions to improve quality of life and prevention.
